# Sterilization of polydimethylsiloxane surface with Chinese herb extract: a new antibiotic mechanism of chlorogenic acid

**DOI:** 10.1038/srep10464

**Published:** 2015-05-21

**Authors:** Song Ren, Ming Wu, Jiayu Guo, Wang Zhang, Xiaohan Liu, Lili Sun, Robert Holyst, Sen Hou, Yongchun Fang, Xizeng Feng

**Affiliations:** 1 Tianjin Medical University Cancer Institute and Hospital, National Clinical Research Center for Cancer, Key Laboratory of Cancer Prevention and Therapy, Tianjin 300071, China; 2 State Key Laboratory of Medicinal Chemical Biology, College of Life Science, Nankai University, Tianjin, 300071, China; 3 Institute of Robotics and Automatic Information Systems, Nankai University, Tianjin 300071, China; 4 Institute of Physical Chemistry Polish Academy of Sciences, Kasprzaka 44/52, 01-224 Warsaw, Poland

## Abstract

Coating of polydimethylsiloxane (PDMS) surface with a traditional Chinese herb extract chlorogenic acid (CA) solves the contemporary problem of sterilization of PDMS surface. The *E. coli* grows slower and has a higher death rate on the CA-coated PDMS surfaces. A smoother morphology of these *E. coli* cell wall is observed by atomic force microscopy (AFM). Unlike the reported mechanism, where CA inhibits bacterial growth by damaging the cell membrane in the bulk solution, we find the CA-coated PDMS surface also decreases the stiffness of the cell wall. A decrease in the Young’s modulus of the cell wall from 3 to 0.8 MPa is reported. Unexpectedly, the CA effect on the swarming ability and the biofilm stability of the bacteria can be still observed, even after they have been removed from the CA environment, indicating a decrease in their resistance to antibiotics for a prolonged time. The CA-coated PDMS surface shows better antibiotic effect against three types of both Gram-positive and Gran-negative bacteria than the gentamicin-coated PDMS surface. Coating of CA on PDMS surface not only solves the problem of sterilization of PDMS surface, but also shines light on the application of Chinese traditional herbs in scientific research.

Polydimethylsiloxane (PDMS) is a widely used material for rapid prototyping of microfluidic devices due to its optical transparence, flexibility, gas permeability and low cost^1^,^2^. Sterilization of PDMS surfaces is the key issue in the fabrication of microfluidic systems for biological and medical applications^3^. To solve the sterilization problem, we successfully applied chlorogenic acid (CA), a water extract from “honeysuckle”, a traditional Chinese herb^4^. Chemically, the CA is a non-toxic ester of cinnamic and quinic acids^5^. The production of a CA coating on a hydrophobic PDMS surface is an energy favorable process, stabilized by a free energy change of ~3 kcal/mol. So far, the process seemed to be so satisfactorily understood and well established, but apparently the story about the traditional Chinese herb solving the contemporary problem of PDMS surface sterilization did not come to an end.

We show that bacteria growing on a CA-coated PDMS surface are still alive, but their biological activities are severely hampered. The difference in the properties of the bacteria that survived and the normal ones cannot be simply characterized by the cell death rate or the cell number. We answer a number of questions: Are the survived bacteria the same as the normal ones? How severely is their vitality hampered? How the cells grow in time on a CA-coated PDMS surface? Our study of changes of the physical and biological properties of the bacteria growing on a CA-coated PDMS surface provides important information for the application of the PDMS surface in clinical and biological fields.

The mechanism of bacterial growth inhibition on a CA-coated PDMS surface is elusive. It has been reported that the CA effects on bacteria consists in damaging the cell membrane in the bulk solution. The minimum inhibition concentration (MIC) of CA for *E. coli* is 80 μg/ml^6^. On the PDMS surface there is only a thin CA layer and therefore the amount of this compound is much smaller than in bulk solution. How can CA still exhibit antibiotic ability in such a small amount? Moreover, the influence of antibiotics on the biological activities of bacteria can be prolonged for a certain period of time after the bacteria have been removed from the environment containing antibiotics, probably due to the time period necessary for adaptation of the bacteria to the new environment^7^. Prolonged CA effect on bacteria after detachment from the surface is important for the sterilization of microfluidic devices in their downstream part. A flow in such a device can take the bacteria from the surface and move them into other microfluidic compartments, where a new colony of bacteria will grow. Unfortunately, no such studies have been carried out so far.

Herein we elucidate whether and how a CA thin layer coated on the PDMS surface inhibits the bacterial growth. As reported, CA exhibits antibiotic effect on a variety of Gram-positive and Gram-negative bacteria. The MIC ranges from 20 to 80 μg/ml depending on the type of bacteria. *E. coli* exhibits the strongest resistance to CA compared to the other bacteria reported to date. (The MIC of *E. coli* is twice as high as that of *Bacillus subtilis*, *Staphylococcus aureus* and *Salmonella typhimurium*, and is four times higher than that of *Streptococcus pneumonia* and *Shigella dysenteriae*^6^.) In this study we chose *E. coli* as a model bacterium because of its highest resistance to antibiotics. We studied the cell growth and death rate of *E. coli* on the CA-coated PDMS surface as a function of culturing time. We observed the morphology of the survived *E. coli* grown on the CA-coated PDMS surface with AFM. We compare them to the *E. coli* grown on the uncoated PDMS surface. A comparison study was also performed on the Young’s modulus of the cell wall, the swarming ability and the biofilm stability of the *E. coli* which grow on both surfaces respectively. The Young’s modulus shows the stiffness of the *E. coli* against the environmental physical pressure. The swarming ability and the biofilm stability of bacteria are related to their resistance to antibiotics and infection abilities of the pathogen bacteria^8^,^9^,^10^.

## Results

### Inhibition of *E. coli* growth on a PDMS surface coated with CA

**Figure 1:**
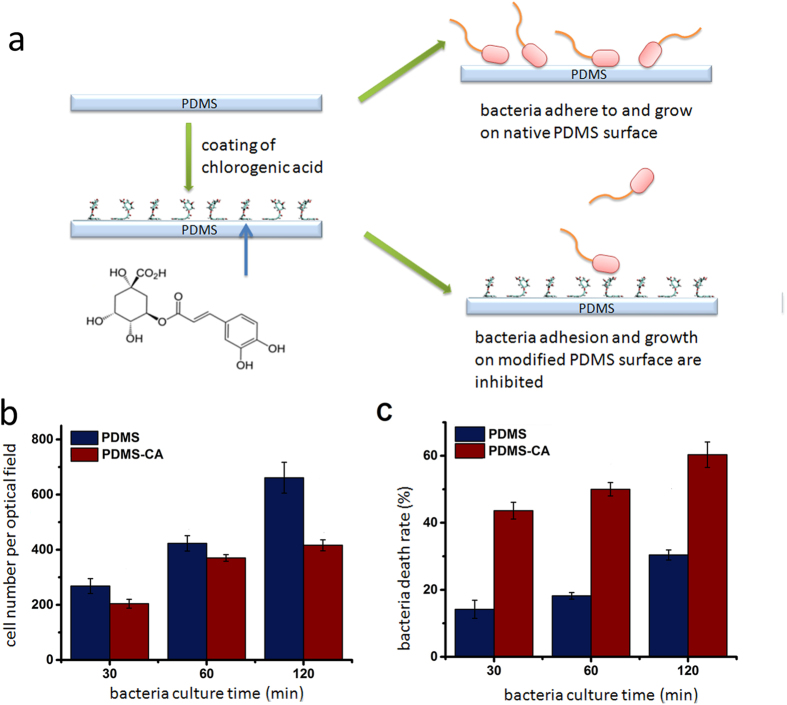
(**a**) Coating of CA for sterilization of PDMS. Chlorogenic acid is the component extracted from a Chinese traditional herb honeysuckle. (**b**) *E. coli* DH5α growth rate on a PDMS surface coated with CA and an uncoated PDMS surface as a function of culturing time. (**c**) *E. coli* DH5α death rate on a PDMS surface coated with CA and an uncoated PDMS surface as a function of culturing time.

CA has been widely used in medical industry, the healthcare industry, cosmetics industry and food industry due to its nontoxic, antibacterial and antiviral properties^5^,^11^. The adsorption of CA onto the PDMS surface is an energy favorable process with the cinnamic acid moiety adsorbing to the PDMS surface, and the quinic acid moiety interacting with the solvent environment^4^ (Fig. 1a). In order to test the antibiotic ability of a CA-coated PDMS surface, we created an environment extremely contaminated with bacteria by immersing the CA-coated PDMS surface in an *E. coli* culture medium, where *E. coli* continuously contaminated the PDMS surface from the bulk culture medium (Fig. 1a). The CA-coated PDMS surface shows a strong ability to inhibit cell growth and a moderate ability to induce cell death (Fig. 1b,c). The *E. coli* grow significantly slower on the CA-coated PDMS surface than on the uncoated PDMS surface. For example, the average number of *E. coli* on the CA-coated PDMS surface is ~60% of that on the uncoated PDMS surface after 2 hours. Since the CA-coated PDMS surface has been carefully rinsed several times to get rid of the loosely attached CA, the antibiotic ability of the CA-coated PDMS surface is probably provided by the attached CA molecules directly contacting with the bacterial surface. This result shows that the CA-coated PDMS surface has a strong antibiotic ability even though the amount of CA, which has been coated on the PDMS surface, is very small. The cell death rate for *E. coli* on a CA-coated PDMS surface is also higher than that for the bacteria on an uncoated PDMS surface. The small amount of CA on the PDMS surface is not only sufficient to affect the growth of *E. coli* (which is shown as the cell number) but also is sufficient to kill a certain part of *E. coli* (which is shown as the death rate) (see the images of *E. coli* in the supplementary information). This result well agrees with the strong antibiotic ability of highly concentrated CA in the bulk solution as reported in a previous study^6^, although the amount of CA used in our study is much lower.

### Morphology changes

**Figure 2:**
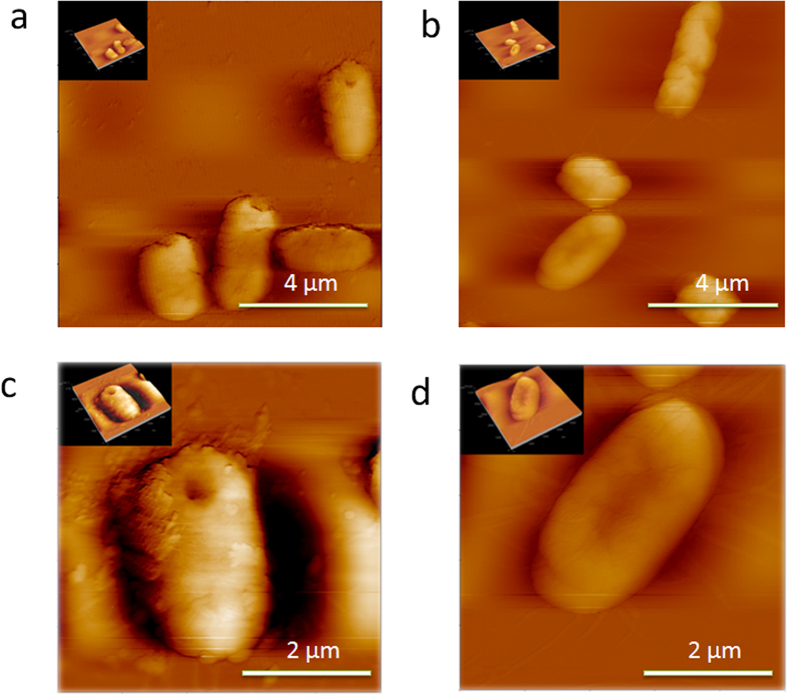
AFM image of a single *E. Coli* DH5α bacterium growing on a native PDMS surface (**a**) and (**c**), and a CA-coated PDMS surface (**b**) and (**d**).

The inhibition of the *E. coli* growth on a CA-coated PDMS surface indicates a change in biological properties of the bacteria. This is confirmed by a difference in the bacteria morphology (Fig. 2). The *E. coli* growing on both the CA-coated and the uncoated PDMS surfaces have a typical rod-like shape of 1–1.5 μm in height and 2–3 μm in length. The size of the *E. coli* agrees with that reported in a previous study^12^. The *E. coli* growing on the CA-coated PDMS surface have a relatively smoother surface. Several coarse speckles on the surface of the *E. coli* growing on the native PDMS surface are observed. They are supposed to be formed by micro-structures of the *E. coli* cell wall. The CA is speculated to affect the surface polysaccharide or the surface protein composition leading to a decrease in a number of flagella. Thus the morphology of the *E. coli* surface becomes smoother. The changes affect the stiffness of the cell wall, the swarming ability and the biofilm stability as shown in the following sections.

### CA on the PDMS surface makes the cell wall fragile

**Figure 3:**
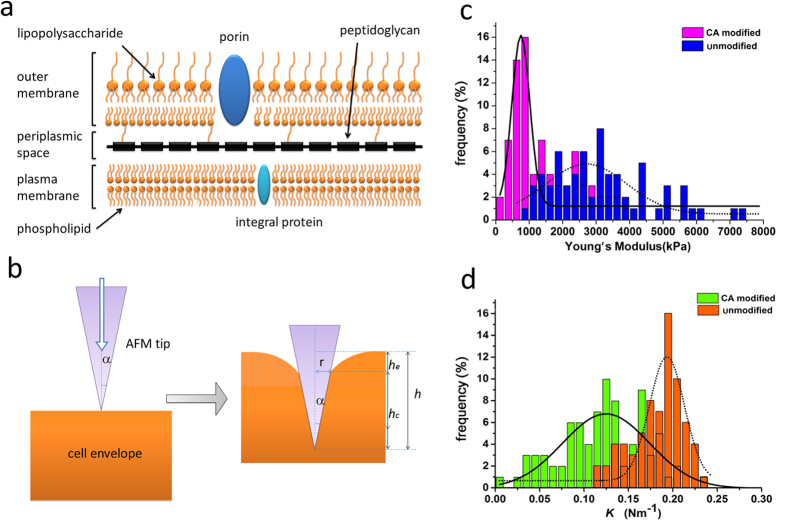
a) Structure of the *E. coli* bacterial cell envelope showing the outer membrane, the peptidoglycan, and the inner plasma cell membrane. **b**) Distortion of the *E. coli* cell wall with a AFM tip indenting inward. **c**) Distribution of the Young’s modulus of the *E. coli* DH5α grown on CA-coated and uncoated PDMS surfaces. **d**) Distribution of the bacterial spring constant *K* of the *E. coli* DH5α growing on CA-coated and uncoated PDMS surfaces.

We quantified the stiffness of the cell wall by measuring the Young’s modulus and the bacterial spring constant. *E. coli* belong to the Gram-negative bacteria, which are classified depending on their ability to be stained by the Gram technique. Unlike the Gram-positive cells, which have a thick cell wall composed of an amorphous matrix of peptidoglycan and secondary polymers, the *E. coli*’s envelope is more complex^13^ (Fig. 3a). The outmost part is a lipid layer containing lipopolysaccharide. The middle layer is a gel-like periplasm and the inner one is a thin peptidoglycan layer. A high turgor pressure is produced inside the periplasm and the cell wall must buoy-up the plasma membrane to protect the bacterial from bursting^14^. When a conical AFM tip approaches the *E. coli* surface, the entire cell envelope is indented with a shape as shown in Fig. 3b. According to Sneddon’s theory, the relation between the force *F* and the indentation distance *h* is
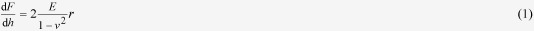
where *v* is the Poisson coefficient which is usually set to 0.5 for biological materials and *r* is the contact radius between the conical AFM tip and the cell envelope. The Young’s modulus *E* is a measure of the stiffness of an elastic material, defined as the ratio of the stress (force per unit area) along an axis over the strain (ratio of deformation over initial length) along that axis in the range of stress in which Hooke’s law holds.

During the indenting process, the shape of the contacting contour keeps changing. The corresponding force-distance curve shows a nonlinear characteristics. The Young’s modulus is fitted from this nonlinear section of the curve and reflects the stiffness of the cell wall. The contacting indentation distance *h*_c_, (the vertical distance from the top of the AFM tip to the contacting point) is a function of the total indentation distance *h*. The relation can be expressed as *h*_c_ = 2*h*/π^15^,^16^. In this case
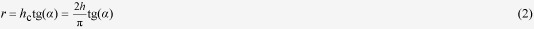
where *α* = 30^o^ is the semi-top angle of the tip.

Integrating Eqs. 1 and 2 we obtain
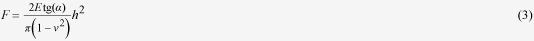


The Young’s modulus *E* is fitted from the force-distance curve with Eq. 3. The Young’s modulus of the *E. coli* growing on an uncoated PDMS surface is ~3 MPa (see the distribution of Young’s modulus in Fig. 3c). This value is the same as that reported by Cerf *et al.*^17^ The *E. coli* growing on a CA-coated PDMS surface has an obviously lower Young’s modulus of ~ 0.8 MPa. The low Young’s modulus of *E. coli* indicates a decrease in the stiffness of the cell wall after the CA treatment. These *E. coli* are supposed to be fragile when subject to both an outer and an inner physical pressure and are prone to cell death.

The increased fragility of the *E. coli* cell wall is verified by measuring the bacterial spring constant *K*. When the AFM tip is further moving into the cell surface, the force-distance curves exhibit as a linear characteristics (see the definition of the linear section and the non linear section in the Methods section). We assume that within this section, the contour contacting area does not shift when the AFM tip indents. The bacterial spring constant *K* is calculated by fitting the force-distance curve with Eq. 4
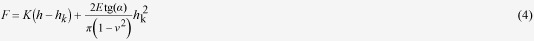
where *h*_k_ is the indentation distance where the linear section of the force-distance curve starts. The *K* value is related to the cell turgor pressure. The cell turgor pressure is the difference between the inner and outer osmotic pressures (higher in cytoplasm), which is the key to keep the shape of cells^18^. The turgor pressure relates to several functions of bacteria including signal transduction, periplasmic transportation, synthesis of porins, to name a few. The *K* value for the *E. coli* grown on a CA-coated PDMS surface is merely a half of those grown on the uncoated PDMS surface. A low *K* value means that the *E. coli* are more likely to be deformed. The value of the bacterial spring constant indicates that the cell turgor pressure of the CA treated *E. coli* is lower than that of the native *E. coli*.

### The CA effect on the *E. coli* continues after the *E. coli* are transferred from the surface into a CA-free environment

We find that the *E. coli* growing on a CA-coated PDMS surface have lower resistance to antibiotics even after they have been removed from a CA environment. The reduced resistance is characterized by decreased swarming ability and biofilm stability.

### Decreased swarming ability

The movement of bacteria over the tops of solid surfaces by using their flagella is called swarming^19^. Swarming mobility is operationally defined as a rapid multi-cellular bacterial movement powered by rotating flagella^20^. It is believed that the swarming ability is related to several bacteriological changes such as the number of flagella per cell, secretion of a surfactant to reduce surface tension for spreading, etc.

**Figure 4:**
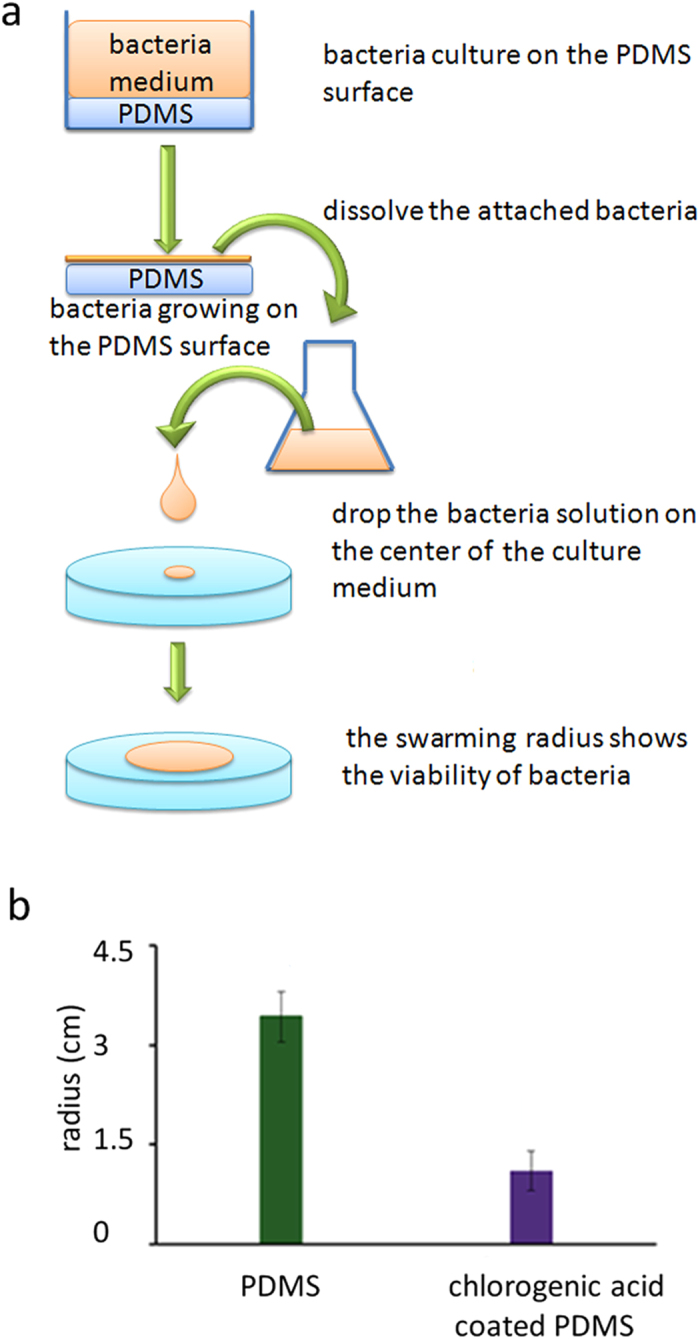
The *E. coli* DH5α harvested from a CA-coated PDMS surface has a lower swarming mobility after transferring onto an agar surface free of CA. **a**) Swarming ability assay of the *E. coli* harvested from a CA-coated PDMS surface and an uncoated PDMS surface. **b**) Swarming radius of the *E. coli* harvested from a CA-coated PDMS surface and an uncoated PDMS surface.

The swarming motility of the *E. coli* harvested from a CA-coated PDMS surface and an uncoated PDMS surface was evaluated by measuring the radius of a bacteria colony on the agar plate (Fig. 4). The radius of the *E. coli* from the uncoated PDMS surface is 3.09 ± 0.38 cm, whereas the radius of bacteria from the CA-coated PDMS surface is 1.63 ± 0.30 cm. Although the *E. coli* were harvested from the CA-coated PDMS surface, their growth on the agar plate was completely free from CA. We think that the CA effect on the *E. coli* still exist for a certain period of time after the *E. coli* have been removed from the PDMS surface coated with CA.

The prolonged swarming lag time is a possible reason for the decreased swarming ability. Bacteria experience a non-motile behavior preceding the initiation of swarming mobility after being transferred from a liquid medium onto a solid surface^21^,^22^. The lag is poorly understood, but an increase in the inoculum density can shorten the lag time^21^. Fig. 1 shows that the *E. coli* grows slower and has a higher death rate on the CA-coated PDMS surface than on the uncoated PDMS surface. After being transferred onto the agar surface, the *E. coli* previously growing on the CA-coated PDMS surface are speculated to maintain the characteristics of slow growth and higher death rate for several generations. *E. coli* need a lag time (ranging from several hours to days) to shift their lifestyle to accommodate to a new environment, a process called adaptation^7^. The slow growth of the *E. coli* harvested from the CA-coated PDMS surface results in a smaller number of bacteria on the swarming agar surface, which further prolongs the swarming lag time. As a result, their swarming ability is weakened.

The difference in the number of flagella on the *E. coli* surface is probably another reason for the difference in the swarming ability. Most bacteria such as *E. coli* have a peritrichous arrangement of flagella contributing to their swarming ability^23^,^24^. The increase of biosynthesis of multiple peritrichous flagella is speculated to be essential for the swarming ability. In the AFM image, the *E. coli* growing on a CA-coated surface show a relatively smoother surface when compared to the *E. coli* growing on the uncoated PDMS surface. We suspect the reason for the smooth surface is that they have less flagella, which results in a decreased swarming ability as shown in Fig. 4.

### Decreased biofilm stability

**Figure 5:**
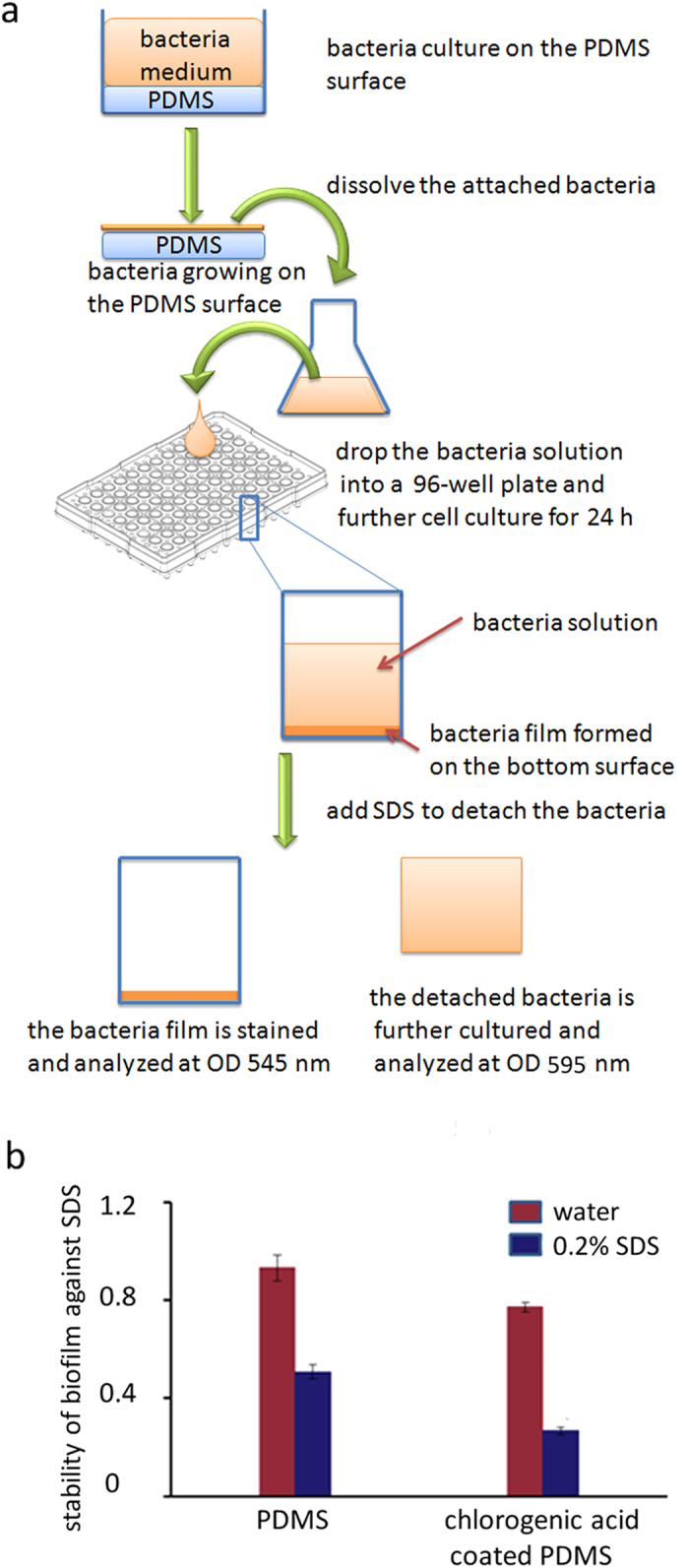
The biofilm formed by the *E. coli* DH5α harvested from a CA-coated PDMS surface has lower stability against SDS than those harvested from the uncoated PDMS surface. The biofilms are formed after the *E. coli* have been transferred outside the CA environment. **a**) Biofilm stability assay against SDS. The number of the *E. coli* in the biofilm and of those released from the biofilm after the SDS treatment were quantified with the absorbance value at 545 nm and 595 nm, respectively. The OD_545nm_/OD_595 nm_ ratio shows the stability of the biofilm against SDS. **b**) The biofilm stability is tested against a 0.2% SDS solution. Water is used as a control for the SDS solution.

Bacteria predominantly grow as multi-species communities attached to submerged surfaces as biofilms^25^,^26^. Biofilms are responsible for 65–80% infections in the human body due to their persistence and chronic nature^27^. An influence of the CA-coated PDMS surface on the biofilm stability is observed. The biofilms are formed after the *E. coli* has been removed from the CA environment. After the treatment with 0.2% SDS, the number of *E. coli* in the biofilm was quantified using the absorbance value at 545 nm. The *E. coli* released from the biofilm was quantified by the absorption value at 595 nm (see details in Methods section). We used the OD_545 nm_/OD_595 nm_ ratio to show the stability of the biofilm against SDS (Fig. 5). Both water and SDS effectively detach bacteria cells from the biofilms. The biofilms formed by the *E. coli* harvested from a CA-coated PDMS surface show a lower stability against SDS than the biofilms formed by the *E. coli* harvested from the uncoated PDMS surface. Specifically, the stability of the biofilms against SDS for the *E. coli* harvested from the CA-coated PDMS surface is less than 50% of the *E. coli* harvested from the uncoated PDMS surface. We suppose that the CA has a long-time effect on the bacteria that survived on the CA-coated surface through a similar adaptation mechanism as discussed for the swarming ability.

### Inhibition of *P. aeruginosa and B. subtilis* growth on a CA-coated and gentamicin-coated PDMS surfaces

**Figure 6:**
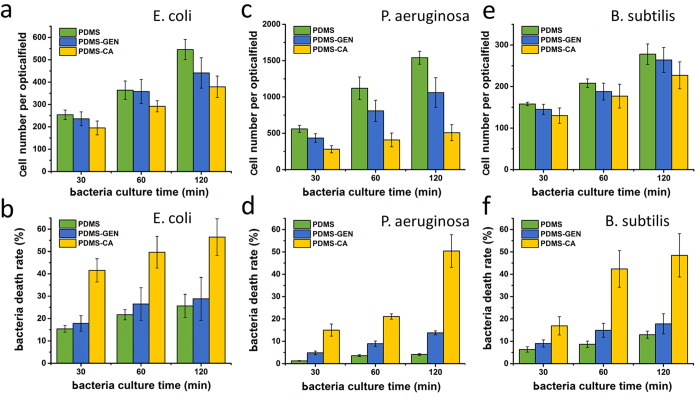
Cell growth rate of **a**) *E. coli* DH5α, **c**) *P. aeruginosa* PAO1 and **e**) *B. subtilis* 168 on the untreated, the gentamicin-coated PDMS and the CA-coated surfaces as a function of culturing time. Bacteria death rate **b**) *E. coli* DH5α, **d**) *P. aeruginosa* PAO1 and **f**) *B. subtilis* 168 on the untreated, the gentamicin-coated and the CA-coated PDMS surfaces as a function of culturing time. The CA-coated PDMS surfaces show a better antibiotic ability than the gentamicin-coated PDMS surfaces.

Gentamicin is a widely used antibiotic. It is effective to treat many types of bacterial infections, particularly those caused by Gram-negative bacteria^28^,^29^. We show that the CA-coated PDMS surface has much stronger antibiotic ability than the gentamicin-coated PDMS surface. *B. subtilis* 168 belongs to Gram-positive bacteria and is not sensitive to gentamicin (see the antibiotic ability of CA and gentamicin to *E. coli* DH5α, *P. aeruginosa* PAO1 and *B. subtilis* 168 in LB solution in the supplementary information Fig. S10). *E. coli* DH5α and *P. aeruginosa* PAO1 are Gram-negative and are more sensitive to gentamicin in LB solution. However CA-coated PDMS surface shows much better antibiotic ability against the Gram-negative bacteria than gentamicin-coated PDMS surface (Fig. 6). A probable explanation is that CA has a cinnamic acid moiety adsorbing to the PDMS surface, which stabilizes the adsorption of CA on PDMS surface by a free energy change of ~3 kcal/mol^4^. Gentamicin, although has a much stronger antibiotic ability against Gram-negative bacteria, is not stable on the PDMS surface. As a result the CA-coated PDMS surface shows much stronger antibiotic ability (see the images of the bacteria in the supplementary information).

## Discussion

The amount of CA on the PDMS surface is much lower than in the bulk liquid at the minimal inhibition concentration. The amount is not sufficient to kill all the *E. coli* bacteria on the PDMS surface. Still, the CA-coated surface is perfect for sterilization applications. In our experiment, the CA-coated PDMS surfaces were exposed to a highly concentrated *E. coli* culture medium. In practice, the concentration of bacteria is usually not so high and the liquid is usually supplemented with certain amount of antibiotics. The *E. coli* shows also the highest resistance to CA compared to all reported 6 kinds of Gram-positive and Gram-negative bacteria^6^. The actual antibiotic ability of the CA-coated PDMS surface should be much better if we use other kinds of bacteria. In addition there is an accidental adsorption of the healthy *E. coli* from the culture medium onto the PDMs surfaces, which results in an underestimation of the sterilization ability.

A new antibiotic mechanism with a small amount of CA is revealed. Previous reports show that the solution medium containing the CA in a concentration higher than MIC can inhibit the *E. coli* growth by damaging the bacterial membrane, which is detected as a leakage of potassium ions, an increase in membrane permeability and a leakage of nucleotides^6^,^30^. We find in this study that besides the damage of the cell membrane, the increased fragility of the cell wall is another possible antibiotic mechanism. During growth, *E. coli* keep colliding with each other and with the container surface due to shaking. The CA treatment makes the *E. coli* fragile to both the outer and inner physical pressure, which finally leads to cell death.

The decrease in the Young’s modulus and the turgor pressure is assumed to be related to the damage of the cell membrane/wall. The cell envelope is supported by the turgor pressure. When the cell membrane is damaged, the cytoplasm components are released. The *E. coli* have lower pressure inside, so the turgor pressure is decreased. Because the cell wall has less support from the cell membrane, it is prone to deformation under the outer pressure.

The decrease of swarming ability and the stability of the biofilm indicates that the CA modified PDMS surface can decrease the *E. coli*’s resistance to antibiotics for a long time. Bacteria of diverse species become resistant to a broad range of antibiotics when swarming^9^,^10^. The mechanism of generalized multidrug resistance seems to be passive owing to rapid spreading of cells at high density^31^. Biofilms protect bacteria from the harsh external environment by producing matrices of extracellular polymeric substances. Formation of biofilms enhances the *E. coli*’s the resistance to antibiotic and biofouling potential^8^, probably because the biofilm cells and the planktonic cells assume distinct lifestyles, which is manifested in both the gene expression level and the sensitivity to ion stress^32^. Although these *E. coli* have already been free of CA, their swarming ability and biofilm stability do not revert to normal immediately. Therefore, further treatment with antibiotics can easily kill these bacteria.

The CA-coated PDMS surface is effective against both Gram-positive and Gram-negative bacteria. This observation expands the application of the CA-coated PDMS surface in the antibiotic field. The antibiotic ability of CA-coated PDMS surface is promoted by the stable adsorption of CA on the PDMS surface, which is stabilized by the cinnamic acid moiety adsorbing to the PDMS surface. Therefore, even though CA does not exhibit antibiotic ability in solution as strong as some traditional antibiotics, such as gentamicin, the CA-coated PDMS surface still exhibits a stronger antibiotic ability than the gentamicin-coated PDMS surface.

## Conclusion

We have successfully applied a Chinese traditional herb extract CA to modify the PDMS surface and have shown that the modified PDMS surface effectively inhibits the *E. coli* growth. The *E. coli* grown on a CA-coated PDMS surface show distinct morphology, lower Young’s modulus, decreased swarming ability and biofilm stability as compared to the *E. coli* grown on an uncoated PDMS surface. A new antibiotic mechanism of the CA is revealed as an ability to decrease the stiffness of the *E. coli* cell wall, which makes these bacteria prone to the cell death caused by a physical damage. Unexpectedly, the CA treatment has a long-time effect on biological properties of the *E. coli*, and the *E. coli* which survive on the CA-coated PDMS surface show a decrease in their swarming ability and biofilm stability even after they have been removed from the CA environment. This result indicates that these *E. coli* have a lower resistance to antibiotics, which is of advantage in the sterilization of microfluidic devices. Even if the bacteria detach from the surface in the devices, their ability to form colonies is highly limited. The CA-coated PDMS surface is also effective against three types of both Gram-positive and Gram-negative bacteria and has a better antibiotic effect than the gentamicin-coated PDMS surface.

## Methods

### Coating of CA and gentamicin on the PDMS surface

PDMS elastomer kit (Sylgard® 184 silicone elastomer kit) was purchased from Dow Corning Corp. Midland, MI. PDMS was produced according to the instructions with a base to curing agent ratio of 10:1. The PDMS was cut into small pieces with a surface area of 1 cm^2^. Chlorogenic acid extracted from honeysuckle (Nanjing Zelang Medical Technology Co. Ltd., China) was dissolved in double-distilled water to a concentration of 10 mg/ml. The PDMS surface was coated with CA solution and incubated at room temperature for 2 h. Then the PDMS surface was washed with double-distilled water to get rid of the unattached CA molecules. The PDMS surface coated with CA was dried with a nitrogen stream (see a scheme of experiment design in Fig. 1a). The successful coating is confirmed by the water contact angle measurement and the X-ray photoelectron spectroscopy (XPS) measurement in our early report^4^. Gentamicin sulfate (Jiangsu Lian Shui Pharmaceutical Co. Ltd., China) was dissolved in PBS to a concentration of 10 mg/ml. The PDMS surface was coated with gentamicin solution and incubated at room temperature for 2 h. The PDMS surface was washed with double-distilled water and then dried with a nitrogen stream.

### Detection of the integrity of *Escherichia coli*, *Pseudomonas aeruginosa*on and *Bacillus subtilis* on PDMS surface

We used *E. coli* DH5α, *P. aeruginosa* PAO1 and *B. subtilis* 168 to study the viability of bacteria on the CA-coated PDMS and untreated PDMS surfaces. Data was measured with a fluorescent microscope (TE 2000-U Nikon, Japan). Gentamicin-coated PDMS surface was used as a positive control. *E. coli* DH5α expressing green fluorescent protein (GFP), *P. aeruginosa* PAO1 and *B. subtilis* 168 were harvested in the exponential growth phase. The CA-coated and gentamicin-coated PDMS and the untreated PDMS were incubated in a bacteria solution at 37 ^o^C for two hours. After incubation, the surfaces were rinsed twice with PBS to get rid of unattached bacteria. The surfaces were stained with a 10 mg/l propidium iodide (PI, Sigma-Aldrich, USA) solution for 15 minutes and then rinsed with double-distilled water to release the extra PI. PI can specifically intercalate into the DNA double helix, but cannot pass the cell membrane. Due to destruction of the cell membrane, PI marked them with red fluorescence.

### Observation of *E. coli* with AFM

AFM images of *E. coli* were recorded with a CSPM4000 atomic force microscope (Ben Yuan Nanometer Instrument Co. Ltd) equipped with silicon nitride cantilevers (CSC21/Cr-Au) of conical shape (Mikro Masch Eesti, Estonia). The force constant is 0.12 N/m. The images were scanned in a tapping mode.

### Measurements of the Young’s modulus of the cell wall with AFM

The Young’s modulus and the turgor pressure were measured by successively approaching the AFM tip toward specified locations of the cell surface. The force curves were recorded with the same AFM equipment mentioned above. In each force curve, 2700 data points were obtained. We measured and averaged the data from all the regions of the cell surface, including the central and the edge parts. We calibrated the sensitivity coefficient of the cantilever and the “Zero” distance. We transformed the force curves into the force-displacement curves and then the force-distance curves (see an example of transition of the force curves to the force-distance curves in the supplementary information). The sensitivity coefficient of the cantilever was calibrated by measuring the force-displacement curves of the AFM tip approaching quartz glass. The quartz glass is a rigid material and is assumed to experience negligible deformation when mechanically contacting with the AFM tip. The slope of the force-displacement curves in the mechanical contact period is the sensitivity coefficient. When the AFM tip is gradually approaching the *E. coli* surface, the force-distance curve exhibits three regimes, 1) electrostatic interactions between the tip and the *E. coli* surface before contacting, 2) mechanical contact between the AFM tip and the *E. coli* surface before deformation of the *E. coli* surface, and 3) mechanical deformation of the *E. Coli* surface by the AFM tip^33^. Regimes 2) and 3) are separated by the “Zero” distance point. “Zero” distance is the flex quantity of piezoelectric ceramic tube when the value of the interaction force in mechanical contact regime is zero. The force-distance curve in regime 3) can be further divided into a non-linear deformation section and a linear deformation section. The Young’s modulus *E* was obtained from fitting of the force-distance curves in the non-linear deformation section. The bacterial spring constant *K* was obtained from fitting of the force-distance curve in the linear section. The distributions of the Young’s modulus and the spring constant were obtained with the method of segmentation of the force-distance curve via the piecewise polynomial approximation^33^.

### Swarming Mobility assay

After two hours of cultivation as indicated above, the culture medium was removed from the CA-coated PDMS and the uncoated PDMS surfaces. The CA-coated and the uncoated PDMS surfaces were waggled in a LB medium solution to detach the bacteria from the surfaces. 10 μl of the harvested *E. coli* solution were dropped into the center of a 0.3% agar plate for swimming assay. Most bacteria require media solidified with agar concentration above 0.3% to force the bacteria to move in the form of swarming but not swimming. Swimming mobility is a similar mode of bacterial movement powered by rotating flagella, but takes place as individual cells moving in liquid environments. The radius of *E. coli* was measured after incubation at 37 ^o^C for 168 h.

### Biofilm stability assay

We use sodium dodecyl sulfate (SDS) to test the stability of the *E. coli* biofilm. The stability of biofilms against SDS was evaluated by the ratio of number of *E. coli* in the biofilm to the number of the detached *E. coli* with a slightly modified a biofilm assay method^34^,^35^. After two hours of cultivation on a CA-coated PDMS surface, the planktonic *E. coli* was removed by rinsing the surface with PBS buffer. The *E. coli* on the surface were harvested by waggling in a LB medium solution. 150 μl of the *E. coli* solution was put into each well of a 96-well plate for further 24 h stationary incubation at 37^ o^C. Then the bacteria medium was replaced by 150 μl of fresh LB medium containing 0.2% of SDS. The mixture was incubated under stationary conditions for 30 min at 37 °C. The suspension of the culture medium was transferred into another well and the number of planktonic *E. coli* was quantified using the absorbance value at 595 nm. The biofilm on the bottom was stained with 1.0% crystal violet for 30 min, washed using deionized water to remove excessive crystal violet and then incubated in a 100% ethanol solution to dissolve the crystal violet bound to the biofilm. The number of *E. coli* in the biofilm was quantified using the absorbance of the crystal violet ethanol solution at 545 nm. An iMark microplate absorbance reader (BioRad, Richmond, CA, USA) was used. The stability of the biofilm was presented as OD_545 nm_/OD_595 nm_. Water was used as a control.

## Additional Information

**How to cite this article**: Ren, S. *et al.* Sterilization of polydimethylsiloxane surface with Chinese herb extract: a new antibiotic mechanism of chlorogenic acid. *Sci. Rep.* **5**, 10464; doi: 10.1038/srep10464 (2015).

## Supplementary Material

Supporting Information Supplementary Figures 1-6
