# Supplementary material for: Sterilization of polydimethylsiloxane surface with Chinese herb extract: a new antibiotic mechanism of chlorogenic acid
Source: Sci Rep. 2015 May 21;5:10464. doi: 10.1038/srep10464 (PMC4650805; doi:10.1038/srep10464)
Supplement: Supporting Information — Supplementary Figures 1-6 [file srep10464-s1.pdf]

Supplementary information for

Sterilization of polydimethylsiloxane surface with a Chinese herb extract: new antibiotic mechanism of chlorogenic acid

Song Ren[1,†], Ming Wu[2,†], Jiayu Guo[3,†], Wang Zhang[2], Xiaohan Liu[3], Lili Sun[4], Robert Holyst[4,*], Sen Hou[2,4,*], Yongchun Fang[3] & Xizeng Feng[2,*]

[1]Tianjin Medical University Cancer Institute and Hospital, National Clinical Research Center for Cancer, Key Laboratory of Cancer Prevention and Therapy, Tianjin 300071, China.

[2]State Key Laboratory of Medicinal Chemical Biology, College of Life Science, Nankai University, Tianjin, 300071, China.

[3]Institute of Robotics and Automatic Information Systems, Nankai University, Tianjin 300071, China.

[4]Institute of Physical Chemistry Polish Academy of Sciences, Kasprzaka 44/52, 01-224 Warsaw, Poland

†These authors contributed equally

Correspondence should be addressed to S.H. (email: hs0010910@gmail.com), R.H. (email: rholyst@ichf.edu.pl), and X.F. (email: xzfeng@nankai.edu.cn)

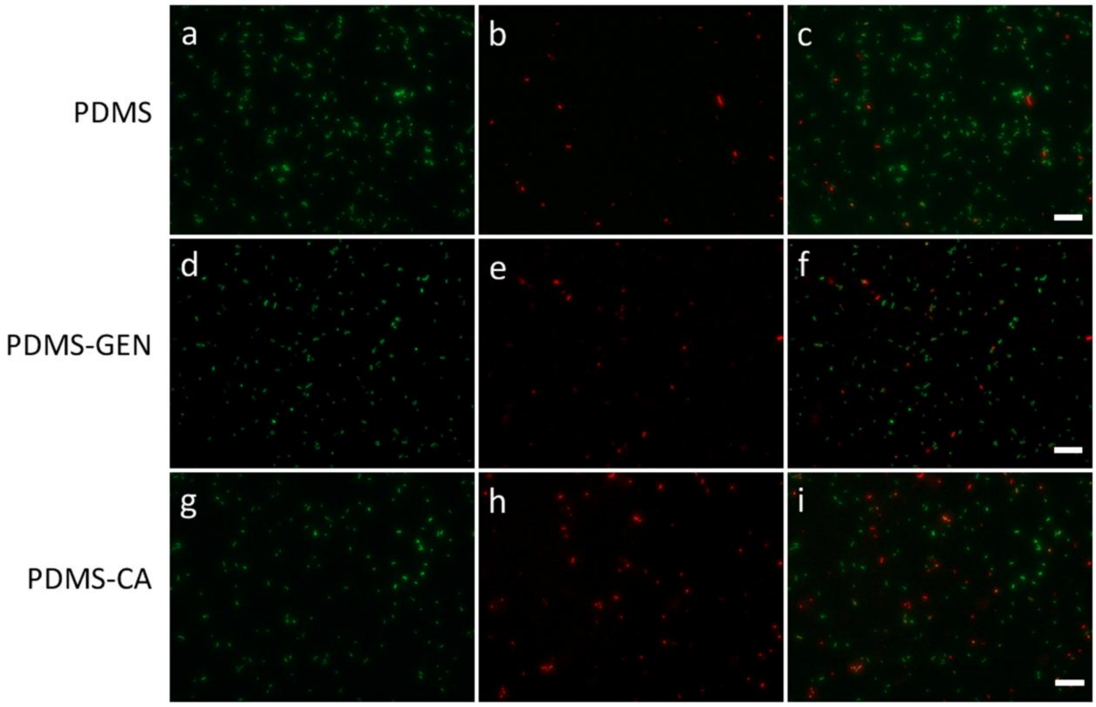

Figure S1. Growth of *E. coli* DH5α on a-c) the uncoated, d-f) the gentamicin-coated and g-i) the CA-coated PDMS surfaces after cell culture for 30 min. a), d) and g) are the fluorescence microscopy images of *E. coli* cells alive. b), e) and h) are the fluorescence microscopy images of dead *E. coli* cells. c) is the merged image for a) and b). f) is the immerged image for d) and e). i) is the immerged image for g) and h). The length of scale bar is 20 μm.

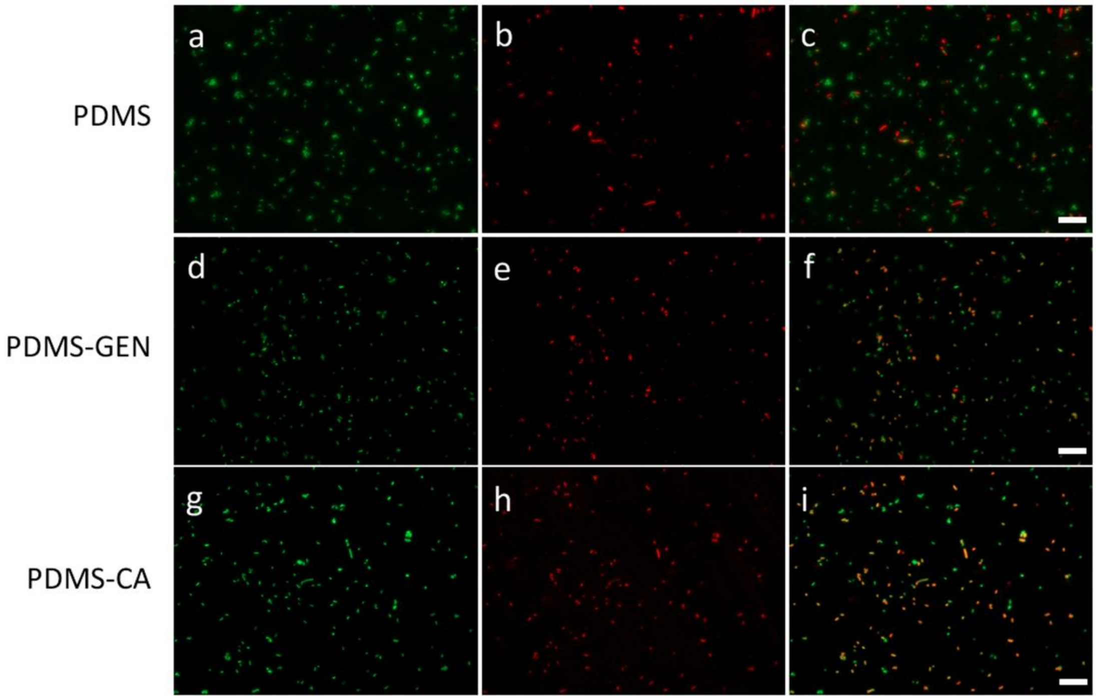

Figure S2. Growth of *E. coli* DH5α on a-c) the uncoated, d-f) the gentamicin-coated and g-i) the CA-coated PDMS surfaces after cell culture for 60 min. a), d) and g) are

the fluorescence microscopy images of *E. coli* cells alive. b), e) and h) are the fluorescence microscopy images of dead *E. coli* cells. c) is the merged image for a) and b). f) is the immerged image for d) and e). i) is the immerged image for g) and h). The length of scale bar is 20 μm.

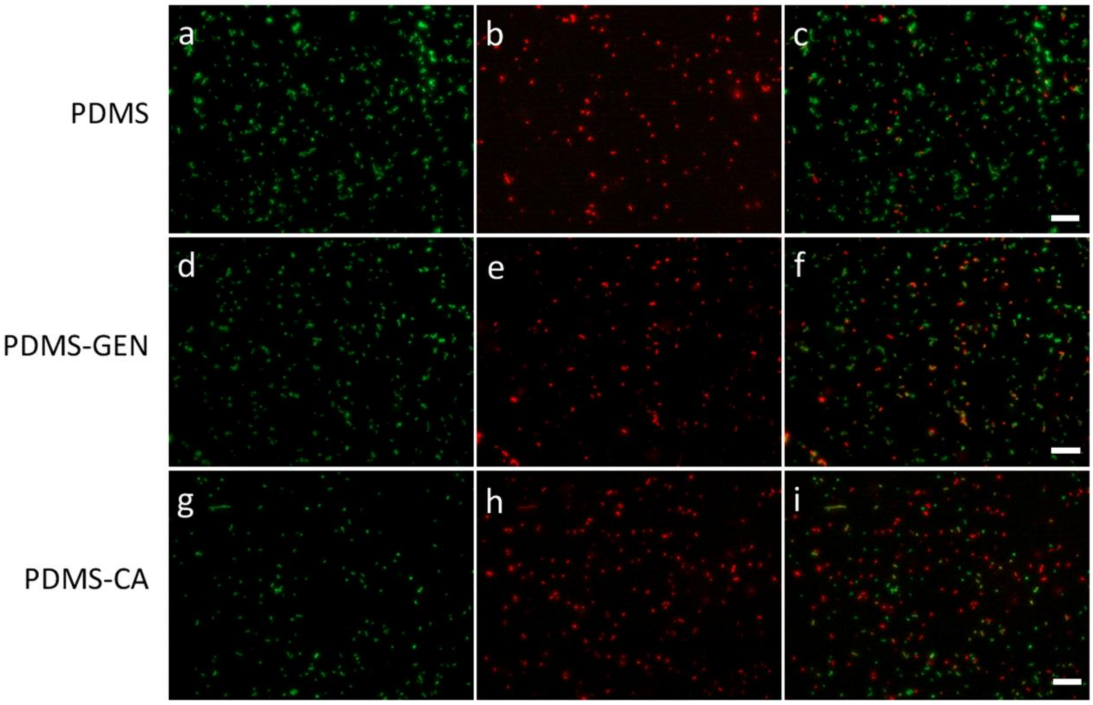

Figure S3. Growth of *E. coli* DH5α on a-c) the uncoated, d-f) the gentamicin-coated and g-i) the CA-coated PDMS surfaces after cell culture for 120 min. a), d) and g) are the fluorescence microscopy images of *E. coli* cells alive. b), e) and h) are the fluorescence microscopy images of dead *E. coli* cells. c) is the merged image for a) and b). f) is the immerged image for d) and e). i) is the immerged image for g) and h). The length of scale bar is 20 μm.

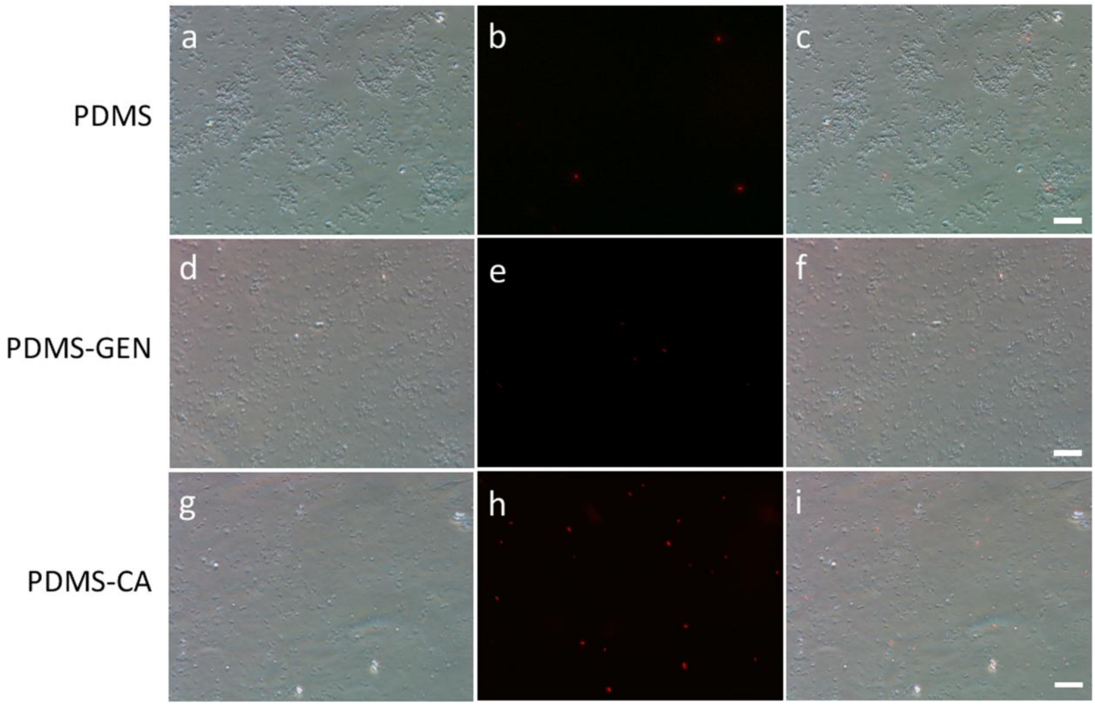

Figure S4. Growth of *P. aeruginosa* PAO1 on a-c) the uncoated, d-f) the gentamicin-coated and g-i) the CA-coated PDMS surfaces after cell culture for 30 min. a), d) and g) are the microscopy images. b), e) and h) are the fluorescence microscopy images of dead bacteria. c) is the merged image for a) and b). f) is the immerged image for d) and e). i) is the immerged image for g) and h). The length of scale bar is 20 μm.

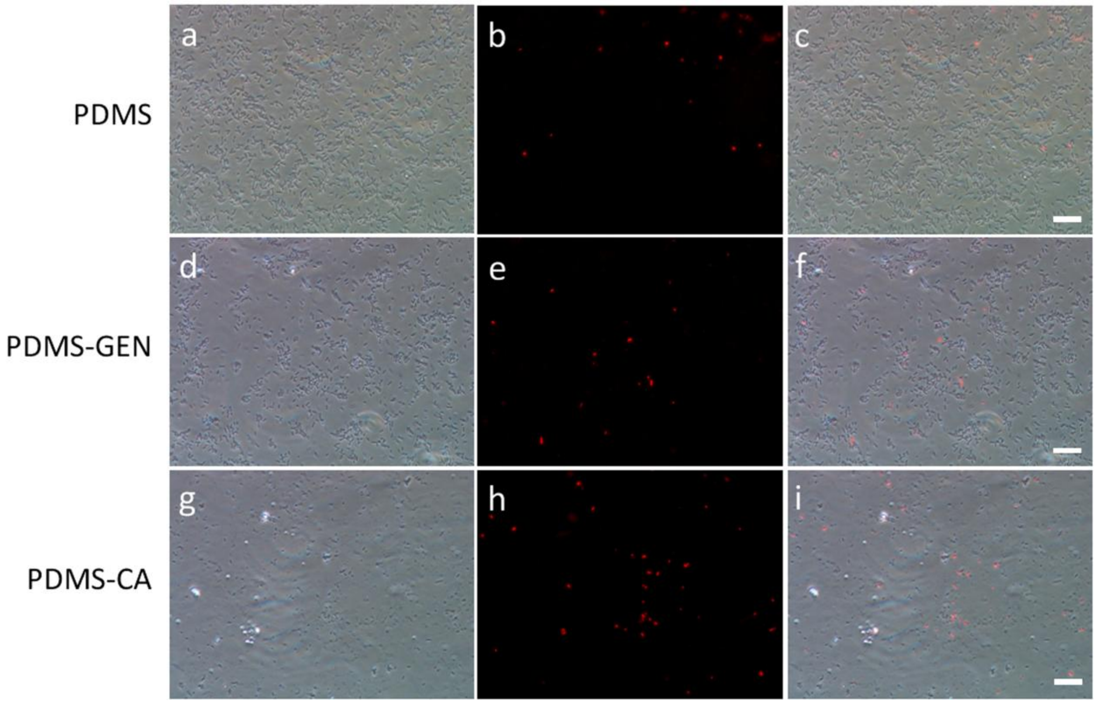

Figure S5. Growth of *P. aeruginosa* PAO1 on a-c) the uncoated, d-f) the

gentamicin-coated and g-i) the CA-coated PDMS surfaces after cell culture for 60 min. a), d) and g) are the microscopy images. b), e) and h) are the fluorescence microscopy images of dead bacteria. c) is the merged image for a) and b). f) is the immerged image for d) and e). i) is the immerged image for g) and h). The length of scale bar is 20 μm.

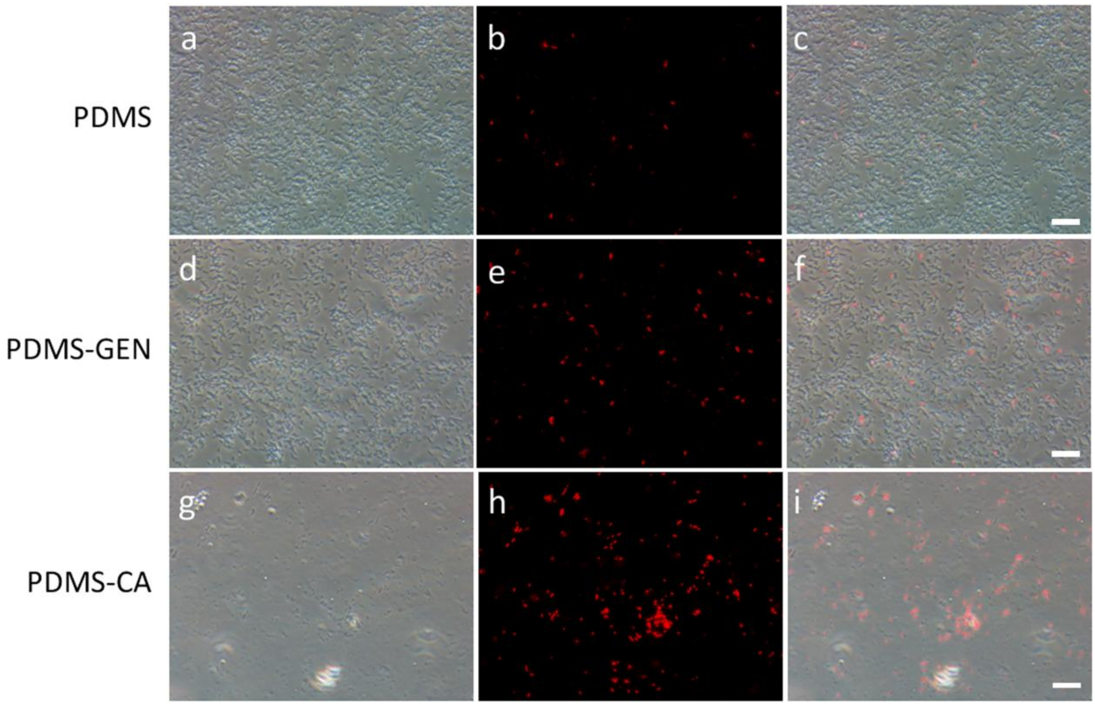

Figure S6. Growth of *P. aeruginosa* PAO1 on a-c) the uncoated, d-f) the gentamicin-coated and g-i) the CA-coated PDMS surfaces after cell culture for 120 min. a), d) and g) are the microscopy images. b), e) and h) are the fluorescence microscopy images of dead bacteria. c) is the merged image for a) and b). f) is the immerged image for d) and e). i) is the immerged image for g) and h). The length of scale bar is 20 μm.

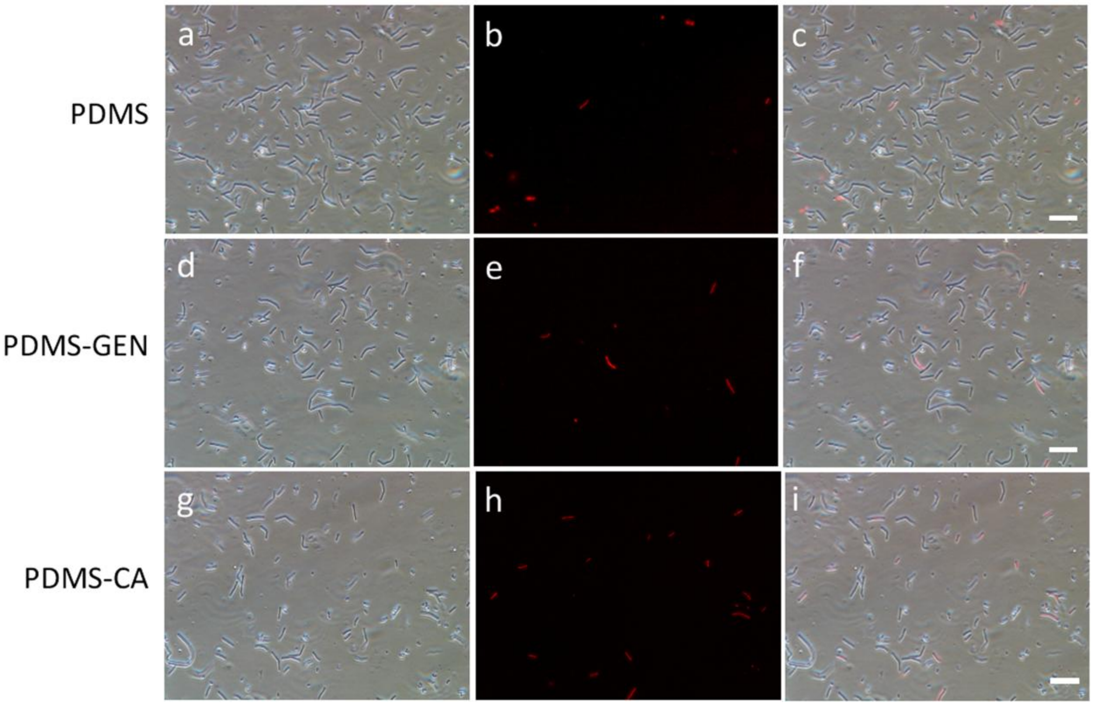

Figure S7. Growth of *B. subtilis* 168 on a-c) the uncoated, d-f) the gentamicin-coated and g-i) the CA-coated PDMS surfaces after cell culture for 30 min. a), d) and g) are the microscopy images. b), e) and h) are the fluorescence microscopy images of dead bacteria. c) is the merged image for a) and b). f) is the immerged image for d) and e). i) is the immerged image for g) and h). The length of scale bar is 20 μm.

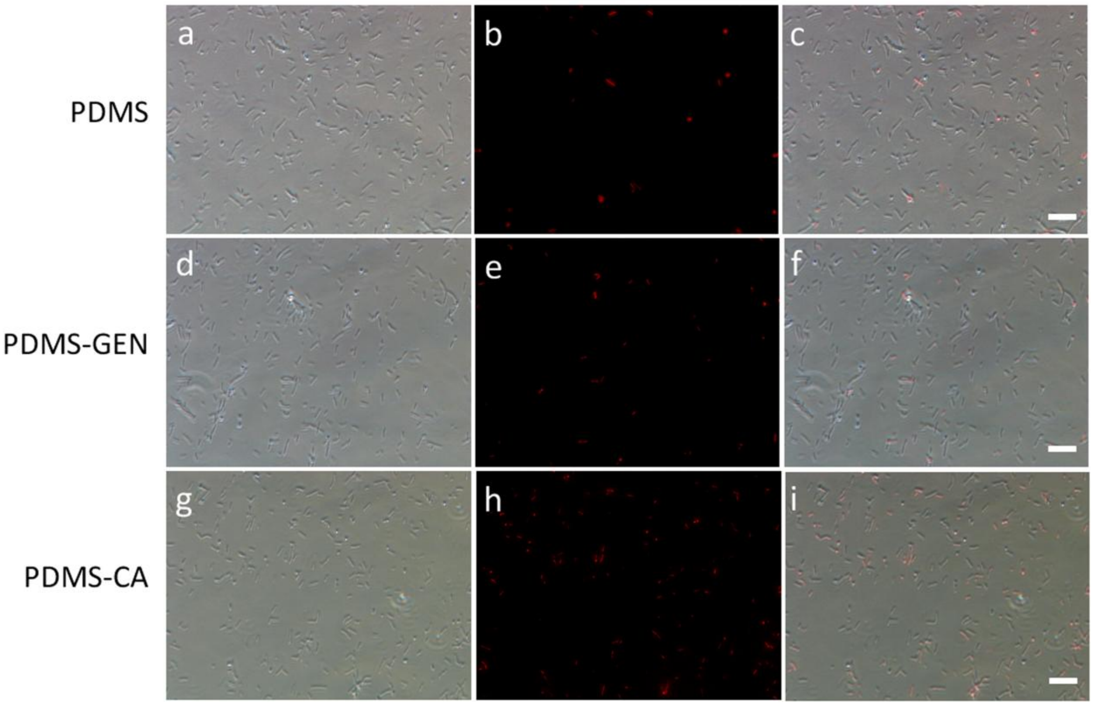

Figure S8. Growth of *B. subtilis* 168 on a-c) the uncoated, d-f) the gentamicin-coated and g-i) the CA-coated PDMS surfaces after cell culture for 60 min. a), d) and g) are the microscopy images. b), e) and h) are the fluorescence microscopy images of dead

bacteria. c) is the merged image for a) and b). f) is the immerged image for d) and e). i) is the immerged image for g) and h). The length of scale bar is 20 μm.

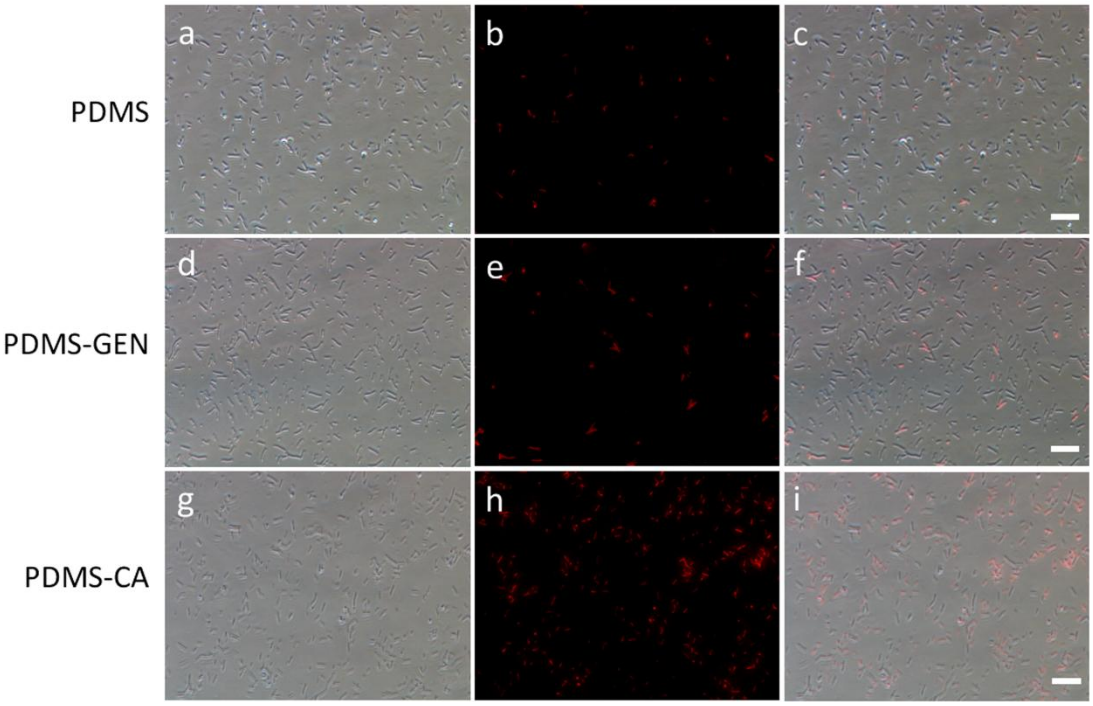

Figure S9. Growth of *B. subtilis* 168 on a-c) the uncoated, d-f) the gentamicin-coated and g-i) the CA-coated PDMS surfaces after cell culture for 120 min. a), d) and g) are the microscopy images. b), e) and h) are the fluorescence microscopy images of dead bacteria. c) is the merged image for a) and b). f) is the immerged image for d) and e). i) is the immerged image for g) and h). The length of scale bar is 20 μm.

Antibiotic ability of CA and gentamicin in LB solution.

The antibiotic ability of CA and gentamicin on *E. coli* DH5α, *P. aeruginosa* PAO1 and *B. subtilis* 168 was tested in LB medium solution. The bacteria were cultured at 37°C for 24 h. The concentration of bacteria was calculated with a hemacytometer chamber (Shanghai Qiu Jing Co. Ltd., China) under a microscope (TE 2000-U Nikon, Japan).

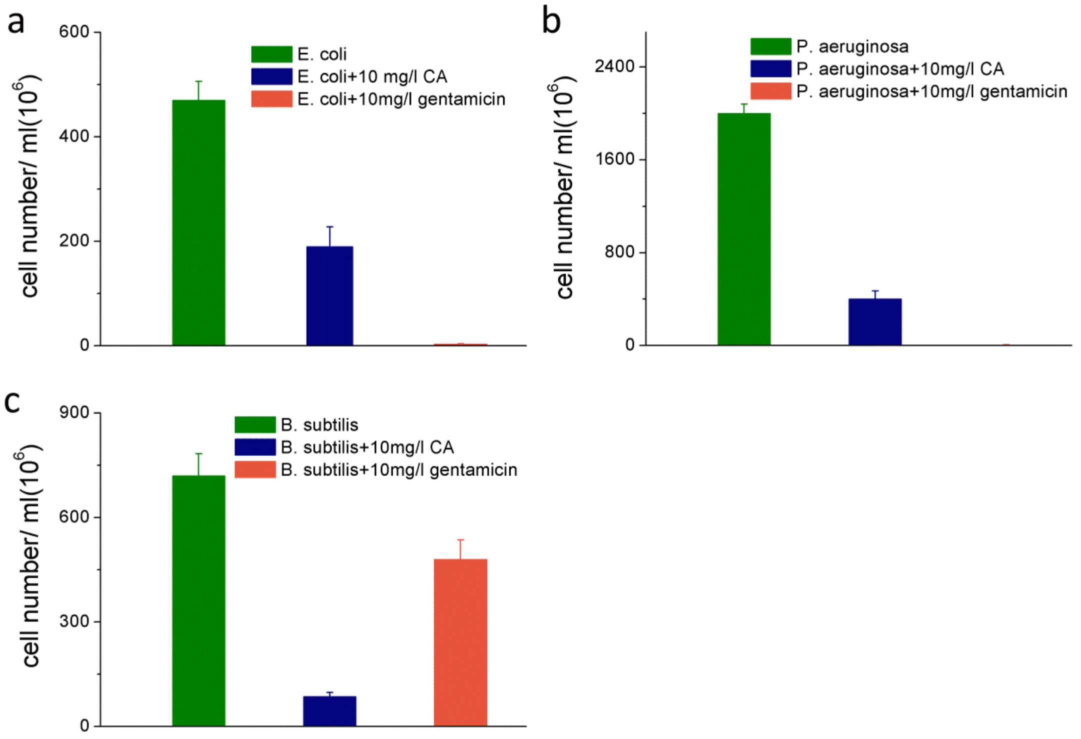

Figure S10. Antibiotic ability of CA and gentamicin in LB solution against a) *E. coli* DH5α, b) *P. aeruginosa* PAO1 and c) *B. subtilis* 168. Gentamicin shows better antibiotic ability than CA against Gram-negative bacteria *E. coli* and *P. aeruginosa*. CA shows a better antibiotic ability against *B. subtilis* than gentamicin.

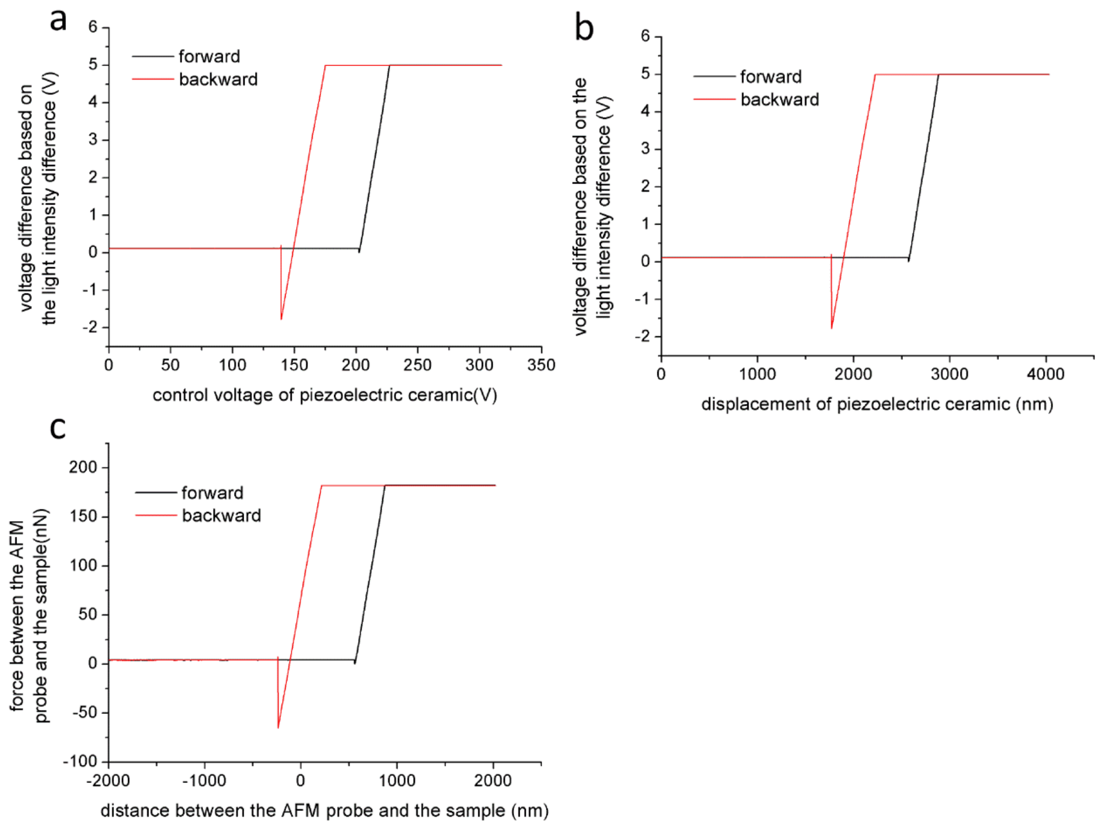

Figure S11. Transition of the force curves to the force-displacement curves and then to the force-distance curves for the measure of Young’s modulus. A rigid quartz glass substrate is used as an example in the measurement. We first get a) the force curves from the AFM equipment. By multiplying the force curves by the expansion coefficient, we could get b) the force-displacement curves. Finally we transform the force-displacement curves to the force-distant curves after calibrating the sensitivity coefficient and “Zero” distance (see the definition in the maintext). The transition of force curves for *E. coli* samples are performed in the same way as done for the quartz substrate.
